# *Mycobacterium chelonae* Infection Identified by Metagenomic Next-Generation Sequencing as the Probable Cause of Acute Contained Rupture of a Biological Composite Graft—A Case Report

**DOI:** 10.3390/ijms23010381

**Published:** 2021-12-29

**Authors:** Andrea C. Büchler, Vladimir Lazarevic, Nadia Gaïa, Myriam Girard, Friedrich Eckstein, Adrian Egli, Sarah Tschudin Sutter, Jacques Schrenzel

**Affiliations:** 1 Division of Infectious Diseases and Hospital Epidemiology, University Hospital Basel, University of Basel, 4031 Basel, Switzerland; a.buchler@erasmusmc.nl (A.C.B.); sarah.tschudin@usb.ch (S.T.S.); 2 Genomic Research Laboratory, Geneva University Hospitals, University of Geneva, 1206 Geneva, Switzerland; vladimir.lazarevic@genomic.ch (V.L.); nadia.gaia@genomic.ch (N.G.); myriam.girard@selexis.com (M.G.); 3 Department of Cardiac Surgery, University Hospital Basel, 4031 Basel, Switzerland; friedrich.eckstein@usb.ch; 4 Clinical Bacteriology and Mycology, University Hospital Basel, 4031 Basel, Switzerland; adrian.egli@usb.ch; 5 Applied Microbiology Research, University of Basel, 4031 Basel, Switzerland; 6 Bacteriology Laboratory, Geneva University Hospitals, 1205 Geneva, Switzerland

**Keywords:** mycobacteria, *Mycobacteroides*, shotgun sequencing, clinical metagenomics, aortic surgery, culture-negative infection

## Abstract

We present the case of a 72-year-old female patient with acute contained rupture of a biological composite graft, 21 months after replacement of the aortic valve and the ascending aorta due to an aortic dissection. Auramine-rhodamine staining of intraoperative biopsies showed acid-fast bacilli, but classical culture and molecular methods failed to identify any organism. Metagenomic analysis indicated infection with *Mycobacterium* *chelonae*, which was confirmed by target-specific qPCR. The complexity of the sample required a customized bioinformatics pipeline, including cleaning steps to remove sequences of human, bovine ad pig origin. Our study underlines the importance of multiple testing to increase the likelihood of pathogen identification in highly complex samples.

## 1. Introduction

Atypical mycobacteria are a rare, but known cause of infectious endocarditis [1]. Case reports and case series reported *Mycobacterium chelonae* (homotypic synonym: *Mycobacteroides chelonae* [2]) as the causative agent of endocarditis associated with foreign bodies or native valves [3,4,5,6].

Metagenomics is an emerging approach for direct analysis of genetic material of mixed microbial communities associated with a host (human, animal, or plant) or an environment (terrestrial or aquatic). In the clinical context, metagenomic next-generation sequencing (mNGS) is used to (i) investigate overall microbiota changes associated with different health conditions, and (ii) to identify pathogens, notably in challenging situations, e.g., when other testing procedures fail. mNGS has the potential to become a routine diagnostic approach, faster than traditional culture-based detection [7]. Provided that a sufficient sequencing depth and bacterial genome coverage are achieved, mNGS may be used for typing [8] and predicting antibiotic resistance [7] of bacterial pathogens.

Here we present the case of a patient with acute contained rupture of a biological composite graft, which illustrates the added value of mNGS in the diagnosis of aortic mycobacterial infections.

## 2. Case Presentation

A 72-year-old female patient presented in deteriorated clinical condition with a reduced state of health, chills, and low diastolic blood pressure for three weeks. The patient had a history of acute aortic dissection Type A twenty-one months earlier. At that time she received aortic root replacement with a biological composite graft consisting of a porcine aortic valve implanted in a tubular, bovine graft (BioIntegral Surgical BioConduit 23 mm, Mississauga, ON, Canada), re-implantation of both coronary ostia, replacement of the distal ascending aorta and proximal aortic arch with a Dacron Graft (Vascutek Gelweave Ante-Flo, Ann Arbor, MI, USA), as well as stenting of the proximal descendant aorta (Gore TAG endostent, Flagstaff, AZ, USA). On present admission, a CT scan showed proximal tear out of the composite annulus as well as the left coronary ostium. Emergency surgery was performed and intraoperatively, a complete destruction and disconnection of the aortic root was witnessed. In all three intraoperative biopsies, acid-fast bacilli were detected using auramin-rhodamin staining (Table 1). Despite the replacement of the aortic graft, the patient died of disseminated intravascular coagulation and multi-organ failure on the following day. At autopsy, necrotizing xantho-granulomatic inflammation was found in the peri-valvular aortic area. Spleen and bone marrow showed no signs of granulomas. All blood cultures remained negative. 

Microbiological work-up showed no evidence of bacterial DNA with broad-range 16S rDNA and other PCR tests in analyzed intraoperative biopsies (Table 1). No growth of bacteria on standard aerobic and anaerobic solid as well as in liquid cultures was found (Table 1). In addition, no mycobacteria were cultured on standard Löwenstein-Jensen nor Bactec MGIT (Becton Dickson, Franklin Lakes, NJ, USA) for 7 weeks at 36 °C, despite the detection of acid-fast bacilli by direct microscopy and the absence of anti-mycobacterial treatment. 

## 3. mNGS and qPCR Analysis

To investigate the discrepancy between the detection of acid-fast bacilli and negative culture, we performed an mNGS analysis of the two intraoperative and one autopsy aorta specimen (Table 1). Each sample was mixed with 100 µL PBS and 150 µL of 10 mg/mL Liberase (TL Research Grade, Sigma-Aldrich, city, state abbreviation, country), incubated for 90 min at 37 °C and passed through a 100 µm-strainer (pluriSelect Life Science, Leipzig, Germany) which was washed twice with 200 µL of the SU buffer from Ultra-Deep Microbiome Prep kit (Molzym, Bremen, Germany). From the obtained suspension, we extracted DNA using an Ultra-Deep Microbiome Prep kit, as per manufacturer recommendations for liquid samples. Negative extraction control (NEC) was performed using the same DNA extraction procedure but omitting the addition of aortic specimens. 

Metagenomic DNA libraries were prepared with Nextera DNA Flex Library Prep Kit (Illumina, San Diego, CA, USA), and sequenced on an Illumina iSeq 100 System with 2 × 151 cycles. Our routine mNGS analysis pipeline (HUGe-MAP) included: (i) read quality filtering with Trimmomatic v0.36 (SLIDINGWINDOW:10:30 MINLEN:100) [9] (ii) removal of replicate sequences (script available at https://github.com/GRL-HUG/duplicates, accessed on 2 August 2021); (iii) removal of read pairs that matched human reference genome build GRCh38 using CLARK v1.2.6.1 (-m 0) [10]; and (iv) classification of read pairs with CLARK (-m 0 -c 0.8) against the collection of Latest RefSeq NCBI [11] reference and representative bacterial, archaeal and fungal genomes, as well as Latest RefSeq NCBI genomes of DNA virus families whose members may infect humans (selected from viralzone.expasy.org, accessed on 31st August 2020). 

The results of HUGe-MAP showed that most reads in the three aorta samples corresponded to the human genome (Table 2). In the surgically obtained aortic prosthesis (Vascutek) sample, 3.92% (100,770) of paired reads remained unclassified. *Mycobacterium* *malmesburyense* (homotypic synonym: *Mycolicibacterium malmesburyense* [2]) was the most abundant microorganism identified by CLARK, being represented by 0.74% (19,943) of all paired reads and by 79.2% of those assigned to bacteria. However, a routine search for 16S rRNA gene fragments with USEARCH v.11.0.667 (with the parameters -id 0.95 -evalue 0.00001 -query_cov 1) [12] against the EzBioCloud 16S rRNA gene sequence database [13] generated 3 hits only, which was about 10-fold less than expected (assuming that 16S rRNA genes roughly correspond to 0.12% of the bacterial genome sequence, as calculated from [14]). Therefore, we selected the reads assigned by CLARK to *M*. *malmesburyense* and queried them against the NCBI genome sequences of bacteria assigned to the genus *Mycolicibacterium* [2] using BLASTN (with the parameters -word_size 15 -evalue 1e-010) [15]. Since no matches were found, we performed an online BLAST analysis of a subset of 100 randomly selected reads against the entire NCBI database, which revealed significant similarities to bovine (*Bos*) sequences. Therefore, we performed a BLASTN analysis (-word_size 15 -evalue 1e-010) of all reads that mapped by CLARK to *M*. *malmesburyense* against the four representative genomes of the *Bos* genus in the NCBI database {*B*. *mutus* (wild yak), *B*. *indicus* × *B*. *taurus* (hybrid cattle), *B*. *taurus* (cattle), and *B*. *indicus* (zebu cattle)}. All reads generated hits, with median (Q1–Q3) sequence identity percentage 99.3 (98.7–100) and median E-value 1.1 × 10^−61^ (1.6 × 10^−70^–4 × 10^−50^). Incidentally, these same reads also mapped to the genomic sequence of *Clostridium botulinum* strain Mfbjulcb6 (NZ_CP027778.1), though with somewhat lower but still substantial similarity {33,641 (84.3%) reads with hits; median sequence identity percentage 94 (88–97.8); median E-value 4.8 × 10^−46^ (6 × 10^−58^–5.2 × 10^−37^)}. The location of all BLASTN hits in the *C*. *botulinum* Mfbjulcb6 chromosomal region representing <1% of the genomic sequence (positions 3,963,803–3,997,481), and the absence of BLASTN hits for other *C*. *botulinum* strains, suggested contamination of the genomic assembly of strain Mfbjulcb6 with sequences of bovine origin.

To reduce misclassification of sequencing reads obtained from the complex aortic samples, we removed the reads corresponding to bovine and pig sequences in addition to those matching the human reference genome, using CLARK-based classification. As reference sequences, we used four *Bos* genomes (see above) and the pig (*Sus scrofa*) genome assembly (GenBank accession GCA_000003025) from the NCBI/RefSeq database. With such a modified bioinformatics pipeline, nearly 4.6% of all paired reads of the aorta prosthesis sample were classified as belonging to genus *Bos*, while the fraction of unclassified and bacterial reads dropped 45-fold and 23-fold, respectively, relative to the original analysis. In the bacterial fraction, *M*. *chelonae* turned out to be the most abundant (Table 2). All the 518 (paired) reads assigned by CLARK to *M*. *chelonae* also were so by BLASTN {median sequence identity percentage 100 (99.3–100); median E-value 2.7 × 10^−69^ (2.7 × 10^−74^–1.6 × 10^−55^)}, and their relative abundance was substantially higher than that of *Cutibacterium acnes*, the organism with highest counts in our NEC. *Moraxella osloensis*, *Micrococcus luteus,* and *Pseudomonas massiliensis*, identified in the aortic prosthesis specimen, also corresponded to putative reagent contaminants. A low percentage of reads associated with *Mycobacterium franklinii* (homotypic synonym: *Mycobacteroides franklinii* [2]) was possibly due to genetic variability of *M*. *chelonae* strains and incompleteness of the reference database used. 

The results of qPCR-based (Qiagen Microbial DNA qPCR Assay for Mycobacterium spp 2, Hilden, Germany) identification of *Mycobacterium abscessus*/*chelonae* complex were in accordance with the NGS data and microscopic examination. These three different tests did not reveal the presence of mycobacteria in the autopsy specimen. In the two intraoperative samples that were NGS- and microscopically-positive for mycobacteria, *M*. *abscessus*/*chelonae* complex was detected by qPCR. Aortic prosthesis sample presented lower Ct and had a higher proportion of bacterial reads assigned to *M*. *chelonae*, as compared to the vegetation.

Culture-based identification of non-pathogenic *Neisseria*, *Enterococcus faecium,* and mixed anaerobic flora in the aortic autopsy specimen (Table 1) was confirmed by mNGS (Table 2).

## 4. Discussion

Our study underlines the importance of multiple testing to increase the likelihood of pathogen identification in highly complex samples. While the classification of k-mers extracted from sequence reads using CLARK (or other similar tools) allows fast processing of millions of reads from metagenomic experiments, it may be advantageous to verify the thus obtained information by read-alignment-based methods, at least on a fraction of reads. This control step may reveal possible artifacts generated by the combination of bioinformatics tools, the choice of reference genomes, and the presence of contaminant sequences in the reference database. It has been recently documented that contamination of prokaryotic genome assemblies with human sequences is particularly problematic in metagenomic analyses, leading to erroneous interpretations [16]. Anecdotally, without prior subtraction of bovine sequences, a substantial fraction of sequencing reads obtained from the aortic prosthesis sample of our patient would be misclassified as belonging to *M. malmesburyense* (using CLARK and NCBI/RefSeq reference/representative genome database) or to *C. botulinum* (using BLASTN and large database consisting of all NCBI/RefSeq bacterial genomes), while the dominant bacterium identified by the modified pipeline (that includes the removal of bovine sequences) was actually *M*. *chelonae*. Intriguingly, in the three samples analyzed, the highest *M.* *chelonae* load was associated with the highest proportion of bovine sequences. Our results suggest a possible origin of *M*. *chelonae* from the transplanted bovine pericardium or porcine aortic valve. Alternatively, the transplanted bovine or porcine tissue favored de novo colonization by *M.* *chelonae*.

## 5. Conclusions

We present the case of a 72-year-old patient with acute contained rupture of a biological composite graft, macroscopic, and histologic signs of an infection due to atypical mycobacteria, 21 months after replacement of the aortic valve and the ascending aorta. The mNGS analysis detected DNA of *M*. *chelonae* as the only potentially causative agent, which was confirmed by specific qPCR. The complexity of the sample required a customized bioinformatics pipeline, including cleaning steps to remove sequences of human, bovine, and pig origin.

In our case, the diagnosis of the infection was complicated by non-conclusive cultures and negative broad-range and species-specific PCRs, despite the detection of acid-fast bacilli using microscopy. Reasons for that might have been the presence of substances in the material obtained by surgery with inhibitory effect on bacterial growth and PCR or contamination with non-living mycobacterial cells. Mycobacteria are a known cause of contamination of foreign material used in cardiovascular surgery [17]. Such contamination may occur via aerosolization arising from contaminated heater-cooler units as has been reported for *M. chimaera* [17], or direct contamination of a prosthesis related to the prosthetic manufacturing process [3]. Detection of the DNA of *M. chelonae* by mNGS analysis suggests a potential cause of the clinical signs in our case. Therefore, we reported this case to the national authorization and supervisory authority for drugs and medical products (Swissmedic Vk_20190123_02).

## Figures and Tables

**Table 1:** Summary of microbiological, molecular and mNGS analyses.

Sample Type and Sampling Time	Microscopy	Culture	PCR	mNGS	*Mycobacterium abscessus*/*chelonae* qPCR Ct *
BioConduit (BioIntegral Surgical) Day 1	Acid-fast bacilli	No growth of mycobacteria or other bacteria	GeneXpert MTB/RIF negative; *Mycobacterium* genus-specific PCR negative; *Mycobacterium chimaera* species-specific PCR negative; Broad-range 16S rDNA PCR negative	ND	ND
Vegetation on aortic prosthesis Day 1	Acid-fast bacilli	No growth of mycobacteria or other bacteria	ND	*M. chelonae* detected below the level of major reagent contaminants	40.12 ± 0.11
Aortic prosthesis (Vascutek) Day 1	Acid-fast bacilli	No growth of mycobacteria or other bacteria	GeneXpert MTB/RIF negative; *Mycobacterium* genus-specific PCR negative; *M. chimaera* species-specific PCR negative; Broad-range 16S rDNA PCR negative	*M. chelonae*	34.06 ± 0.12
Mediastinal swab Day 2	ND	No growth of mycobacteria or other bacteria	ND	ND	ND
Aortic autopsy Day 3	No detection	*Enterococcus faecium* sporadically; apathogenic *Neisseria* spp. 1 CFU; anaerobic mixed flora sporadically; no growth of mycobacteria	ND	No detection	>42

* The volume of DNA extract used was adjusted so to correspond to 38 mg of the frozen specimen mass. Mean ± standard deviation from triplicates are given. Positive PCR control performed using an artificial DNA sequence and corresponding primers showed no signs of inhibition of the qPCR in tested specimens (Ct 20 ± 0.2 for each sample). ND, not done; Ct, cycle threshold.

**Table 2:** Number of read pairs obtained by mNGS) and their taxonomic assignments at various steps of data analysis. NCBI taxonomy was used for CLARK-based species assignments. In this taxonomy, some species of the genus *Mycobacterium* were reassigned to the four new genera including *Mycobacteroides* and *Mycolicibacterium*. In the bacterial fraction, species with the relative abundance of reads >2% are presented. Several species of interest in the NEC under this threshold are also indicated. Yellow background: routine pipeline, blue background: modified pipeline.

Read Pairs	Aortic Prosthesis				Vegetation on Aortic Prosthesis				Aortic Autopsy				NEC			
Raw	5,854,406				4,788,163				5,689,196				32,153			
Quality-filtered	2,699,047				2,823,668				1,579,879				2128			
Human	2,572,953				2,822,051				1,576,184				342			
Bovine	NA		122,725		NA		52		NA		156		NA		1	
Pig	NA		31		NA		11		NA		653		NA		2	
Fungi	136		37		28		25		104		95		13		12	
DNA viruses	0		0		0		0		0		0		0		0	
Unclassified	100,770		2213		1390		1330		3229		2427		934		932	
Bacteria/Archaea	25,188		1088		201		199		365		364		839		839	
Percentage in bacterial/archaeal fraction	*Mycolicibacterium malmesburyense*	79	*Mycobacteroides chelonae*	48	*Cutibacterium acnes*	23	*Cutibacterium acnes*	24	*Rothia mucilaginosa*	7.7	*Rothia mucilaginosa*	6.1	*Cutibacterium acnes*	21	*Cutibacterium acnes*	21
	*Anaerobutyricum hallii*	8.2	*Cutibacterium acnes*	4.9	*Moraxella osloensis*	8	*Moraxella osloensis*	8	*Blautia obeum*	6	*Blautia obeum*	4.8	*Sphingomonas echinoides*	8.6	*Sphingomonas echinoides*	8.6
	*Acetanaerobacterium elongatum*	3	*Mycobacteroides franklinii*	4.2	*Arcobacter lekithochrous*	6.5	*Arcobacter lekithochrous*	6.5	*Gemmiger formicilis*	4.9	*Gemmiger formicilis*	3.9	*Micrococcus luteus*	7	*Micrococcus luteus*	7
	*Catenibacterium mitsuokai*	2.9	*Moraxella osloensis*	4	*Sphingomonas echinoides*	5	*Sphingomonas echinoides*	5	*Streptococcus salivarius*	4.7	*Streptococcus salivarius*	3.7	*Cloacibacterium normanense*	4.1	*Cloacibacterium normanense*	4.1
	*Staphylococcus simiae*	2.2	*Micrococcus luteus*	3.8	*Micrococcus luteus*	4.5	*Micrococcus luteus*	4.5	*Neisseria mucosa*	4.1	*Neisseria mucosa*	3.3	*Acinetobacter johnsonii*	3.6	*Acinetobacter johnsonii*	3.6
	*Mycobacteroides chelonae*	2.1	*Pseudomonas massiliensis*	2.1	*Mycobacteroides chelonae*	2.5	*Mycobacteroides chelonae*	2.5	*Arcobacter lekithochrous*	4.1	*Arcobacter lekithochrous*	3.3	*Enterococcus cecorum*	2.5	*Enterococcus cecorum*	2.5
			*Modestobacter marinus*	2	*Alcanivorax hongdengensis*	2.5	*Alcanivorax hongdengensis*	2.5	*Akkermansia muciniphila*	3.3	*Akkermansia muciniphila*	2.6	*Ralstonia pickettii*	2.2	*Ralstonia pickettii*	2.2
					*Halomonas muralis*	2.5	*Halomonas muralis*	2.5	*Micrococcus luteus*	3	*Micrococcus luteus*	2.4	*Pseudomonas massiliensis*	<2	*Pseudomonas massiliensis*	<2
					*Acinetobacter johnsonii*	2	*Acinetobacter johnsonii*	2	*Enterococcus faecium*	3	*Enterococcus faecium*	2.4	*Halomonas muralis*	<2	*Halomonas muralis*	<2
									*Ruminococcus faecis*	3	*Ruminococcus faecis*	2.4	*Moraxella osloensis*	<2	*Moraxella osloensis*	<2
									*Bacteroides vulgatus*	2.7	*Bacteroides vulgatus*	2.2				
									*Streptococcus parasanguinis*	2.7	*Streptococcus parasanguinis*	2.2				
									*Alistipes inops*	2.2						
									*Fusicatenibacter saccharivorans*	2.2						
Pipeline	Routine		Modified		Routine		Modified		Routine		Modified		Routine		Modified	

NEC, negative extraction control; NA, not applicable.

## Data Availability

After filtering out reads matching human genome sequence, sequencing data were submitted to the European Nucleotide Archive (ENA; www.ebi.ac.uk/ena) under study number PRJEB39759.
